# Hyperlipidaemia in diabetes: are there particular considerations for next-generation therapies?

**DOI:** 10.1007/s00125-024-06100-z

**Published:** 2024-02-20

**Authors:** Sophie Béliard, Florian Mourre, René Valéro

**Affiliations:** 1 APHM (Assistance Publique-Hôpitaux de Marseille), Department of Nutrition, Metabolic Diseases, Endocrinology, La Conception Hospital, Marseille, France; 2 Inserm, INRAE (Institut National de Recherche pour l’agriculture, l’Alimentation et l’Environnement), C2VN (Centre de recherche en CardioVasculaire et Nutrition), Aix Marseille University, Marseille, France

**Keywords:** ANGPTL3, ApoC-III, Cardiovascular disease, Diabetes, HDL-cholesterol, LDL-cholesterol, Lp(a), PCSK9, Review, Therapy, Triglycerides

## Abstract

Dyslipidaemias are major cardiovascular risk factors, especially in people with diabetes. In this area, next-generation therapies targeting circulating lipoparticle metabolism (LDL, VLDL, chylomicrons, HDL) have recently been approved by the European and US medical agencies, including anti- proprotein convertase subtilisin/kexin 9 (PCSK9) antibodies; an siRNA targeting PCSK9; bempedoic acid, which targets ATP citrate lyase; an antisense oligonucleotide targeting apolipoprotein C-III; an anti-angiopoietin-like 3 antibody; and a purified omega-3 fatty acid, icosapent ethyl. Other therapies are in different phases of development. There are several important considerations concerning the link between these new lipid-lowering therapies and diabetes. First, since concerns were first raised in 2008 about an increased risk of new-onset diabetes mellitus (NODM) with intensive statin treatment, each new lipid-lowering therapy is being evaluated for its associated risk of NODM, particularly in individuals with prediabetes (impaired fasting glucose and/or impaired glucose tolerance). Second, people with diabetes represent a large proportion of those at high or very high cardiovascular risk in whom these lipid-lowering drugs are currently, or will be, prescribed. Thus, the efficacy of these drugs in subgroups with diabetes should also be closely considered, as well as any potential effects on glycaemic control. In this review, we describe the efficacy of next-generation therapies targeting lipoprotein metabolism in subgroups of people with diabetes and their effects on glycaemic control in individuals with diabetes and prediabetes and in normoglycaemic individuals.

**Graphical Abstract:**

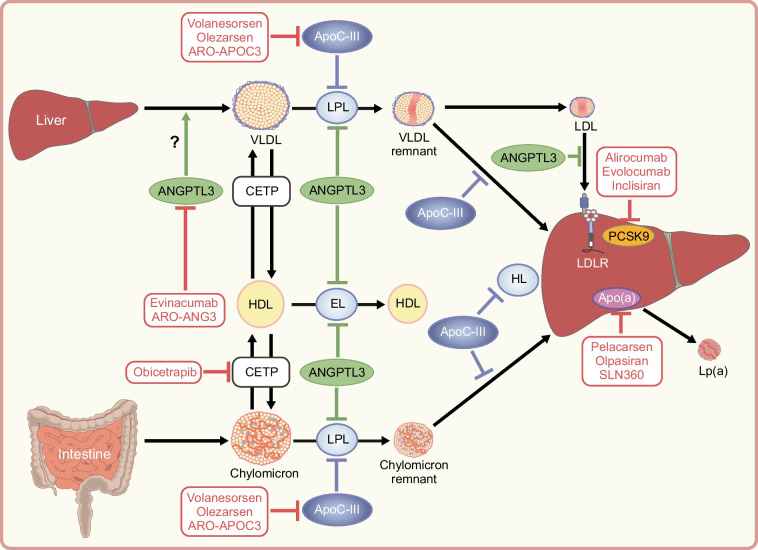

**Supplementary Information:**

The online version contains a slide of the figure for download available at 10.1007/s00125-024-06100-z.


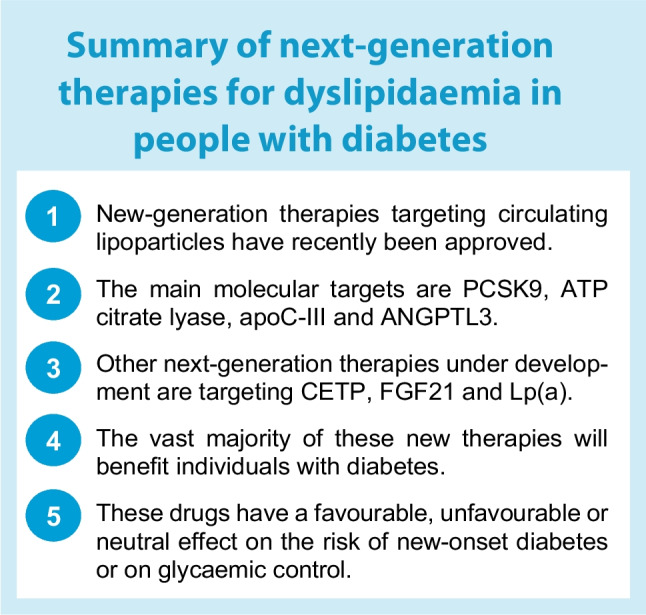


## Introduction

There is a high burden of atherosclerotic CVD (ASCVD) risk in people with diabetes. The primary target to reduce this risk is lowering LDL-cholesterol (LDL-C), with statins the cornerstone of ASCVD prevention [1]. Statins have been available since the late 1980s, but it was only in 2008 that the first alert on the risk of new-onset diabetes mellitus (NODM) under statin therapy was given in the JUPITER study of rosuvastatin [2]. This alert was confirmed in other trials and large meta-analyses, leading the US Food and Drug Administration (FDA) to impose a warning in 2012 regarding glycaemic control in all individuals treated with statins. However, the risk of NODM in those receiving statins is low and is largely outweighed by the cardiovascular benefits [3, 4]. Since this alert, all lipid-lowering therapies have been closely examined for their potential to interfere with glucose metabolism. Beyond LDL-C, the residual cardiovascular risk, which is defined as the risk of cardiovascular events that persists despite achievement of treatment goals for LDL-C, blood pressure and glycaemia, is very prevalent in those with type 2 diabetes [5]. In type 2 diabetes, this risk is often associated with atherogenic dyslipidaemia. Atherogenic dyslipidaemia is mainly characterised by fasting and postprandial hypertriglyceridaemia, a decrease in HDL-cholesterol (HDL-C) and an increase in small and dense LDL particles [6]. The pathophysiology of atherogenic dyslipidaemia is widely explained by the accumulation in blood of triglyceride-rich lipoproteins (TRLs) synthesised by the liver (VLDL) and the intestine (chylomicrons). There are extensive epidemiological, genetic and biological data showing that the increase in TRLs is a causal risk factor for atherosclerosis through direct and indirect mechanisms [7]. However, efficient lipid-lowering therapies targeting atherogenic dyslipidaemia in diabetes are currently missing.

**Fig. 1:**
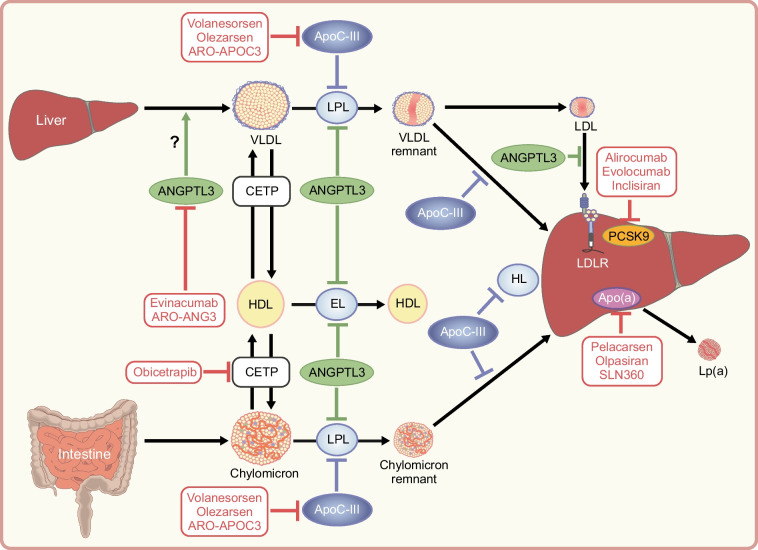
Targets of newly approved and potential future therapies for reducing TRL, LDL-C and lipoprotein (a) (Lp(a)) levels and increasing HDL-C levels. ApoC-III (blue) inhibits LPL and hepatic lipase (HL) and prevents the uptake of TRLs and their remnants by the liver. ANGPTL3 (green) may increase VLDL secretion, inhibits LPL and endothelial lipase (EL) and prevents the uptake of LDL in the liver. PCSK9 (orange) promotes LDL receptor degradation in lysosomes of hepatocytes. CETP (white) promotes the exchange of triglycerides and cholesteryl esters from HDL to apoB-containing lipoproteins. Newly approved and potential future therapies are shown in red. ANGPTL3, angiopoietin-like 3; Apo, apolipoprotein; CETP, cholesteryl ester transfer protein; LDLR, low-density lipoprotein receptor; LPL, lipoprotein lipase; PCSK9, proprotein convertase subtilisin/kexin 9. This figure is available as a downloadable slide

Over the last 10 years, a large number of next-generation therapies in the field of lipidology have been developed, the majority of which will benefit those with diabetes. In this review, we provide an update on these next-generation lipid-modifying therapies, with particular emphasis on their efficacy in the population with diabetes and their potential impact on glucose metabolism. The effects of the newly approved drugs targeting lipoproteins on lipids and glycaemia are summarised in Table 1. The molecular targets of these new therapies (newly approved and potential future therapies) are illustrated in Fig. 1.

**Table 1:** Effects of newly approved drugs targeting lipoproteins on glycaemic and lipid variables

Drug	Effects on lipids					Effects on glycaemia		Ref
	LDL-C	TG	HDL-C	ApoB	Lp(a)	NODM	Glycaemic control in diabetic individuals	
Mainly targeting LDL-C								
Targeting PCSK9								
Alirocumab/evolocumab (antibodies)	Up to −65%	Up to −17%	Up to +9%	Up to −47%	Up to −27%	No	No change	[20–24]
Inclisiran (siRNA)	Up to −51%	Up to −14%	Up to +9%	Up to −36%	Up to −25%			[25–29]
Targeting ATP citrate lyase								
Bempedoic acid	Up to −29%	Up to −5%	Up to −6%	Up to −18%		No	Modestly improved	[30–33]
Mainly targeting TG/HDL-C								
Targeting omega-3 fatty acids								
Highly purified IPE	−7%	−14%	−3%	−7%				[48]
Targeting apoC-III								
Volanesorsen (ASO)	Up to +96%	Up to −88%	Up to +61%	Up to +6%				[52, 54]
Targeting ANGPTL3								
Evinacumab (antibodies)	Up to −25%	Up to −88%	Up to −28%	Up to −40%				[55]

The data represent mean or median change from baseline

ANGPTL3, angiopoietin-like 3; Apo, apolipoprotein; ASO, antisense oligonucleotide; IPE, icosapent ethyl; Lp(a), lipoprotein (a); PCSK9, proprotein convertase subtilisin/kexin 9; TG, triglyceride

## Therapies mainly targeting LDL-cholesterol

### Historical treatments

Statins are the first-line LDL-C-lowering therapy. A prospective meta-analysis of individuals with diabetes showed a reduction of 9% in all-cause mortality, 13% in vascular mortality, 21% in major vascular events, 22% in myocardial infarction (MI) or coronary death, 25% in coronary revascularisation and 21% in stroke per 1.0 mmol/l reduction in LDL-C in the statin therapy group compared with the placebo group [8]. Statins are associated with a low risk of NODM but this does not offset their substantial CV benefits: one extra case of NODM occurred for 1000 person-years of statin exposure while preventing five CV deaths [9, 10]. The exact mechanisms responsible for statin-induced diabetes are currently unknown [11, 12]. Several hypotheses, elaborated from in vivo or animal experiments, have been proposed, with some based on impaired insulin secretion (induced by modification of the cholesterol content of pancreatic beta cells or a direct effect of statins on calcium channels or mitochondria in beta cells) and others based on impaired insulin sensitivity (induced by a decrease in glucose uptake in skeletal muscle cells or alterations in adipocyte differentiation) [13]. A recent and timely study using gold standard tests (insulin infusion and graded glucose infusion tests) showed an increase in insulin resistance (but also an increase in insulin secretion) in those exposed to atorvastatin [14]. Other studies based on Mendelian randomisation or clinical observations also support a role for the LDL receptor (the major actor in the cholesterol-lowering effect of statins) in modest weight gain and/or insulin resistance [15]. Recent results are therefore in favour of an insulin resistance effect induced by statins; however, the cellular mechanisms still need to be elucidated. It was initially thought that this effect was a drug class effect [11]; however, it has been shown that pitavastatin, a more recently introduced statin, is not associated with NODM [16]. Pitavastatin has been recommended over other statins in those at high risk of diabetes, with prediabetes (impaired fasting glucose and/or impaired glucose tolerance) or even with diabetes [17]. It has also been shown that ezetimibe, a second-line LDL-C-lowering therapy, used in association with statins when LDL-C is not at goal or when statins are contraindicated, has no associated risk of NODM [18]. Furthermore, the IMPROVE-IT study showed that ezetimibe in association with simvastatin was more effective at CVD prevention in the subgroup of people with diabetes than in the subgroup without diabetes [19].

### Newly approved treatments

#### Therapy targeting proprotein convertase subtilisin/kexin 9

Proprotein convertase subtilisin/kexin 9 (PCSK9) plays a pivotal role in cholesterol homeostasis by promoting LDL receptor degradation in lysosomes of hepatocytes. The inhibition of PCSK9 results in an increased number of LDL receptors at the hepatocyte membrane, leading to enhanced catabolism of circulating LDL particles (Fig. 1). Several types of drugs that inhibit PCSK9 have been developed over the last 20 years: antibodies, adnectins, siRNAs and even a vaccine. Two PCSK9 antibodies, alirocumab and evolocumab, are efficient lipid-lowering drugs, decreasing LDL-C by 50–65%, triglycerides (TGs) by 15–17% and non-HDL-C by 50–54%, with a similar effect between the two drugs [20, 21] (Table 1). Mendelian randomisation studies have found that genetic variants mimicking the effects of PCSK9 inhibitors are associated with an increased risk of diabetes or hyperglycaemia [22]. These genetic findings have raised concerns about the risk of NODM with the use of PCSK9 inhibitors. A prespecified analysis of FOURIER, a cardiovascular outcomes trial (CVOT) comparing evolocumab with placebo, was carried out to investigate the efficacy of evolocumab in people with diabetes and the associated risk of NODM and the effect of the drug on glycaemic control [21]. This study included 27,564 participants with atherosclerotic disease and treated with statins. Evolocumab demonstrated a similar efficacy in terms of decreasing the primary endpoint (composite of CV death, MI, stroke, hospital admission for unstable angina or coronary revascularisation) in the 11,031 participants with diabetes and in the 16,533 participants without diabetes (RR 0.83 and 0.87, respectively). Evolocumab exposure was not associated with NODM even in those with prediabetes (*n*=10 344), nor with deterioration of glycaemic control in those with diabetes, at a median follow-up of 2.2 years. The similar prespecified ODYSSEY study of alirocumab was published 2 years later. This study included 18,924 participants who had acute coronary syndrome 1–12 months before randomisation and who were treated with high-intensity statins [20]. Alirocumab was equally as effective at reducing CV events (e.g. death from CHD, non-fatal MI, fatal or non-fatal ischaemic stroke or unstable angina requiring hospital admission) in those with diabetes (*n*=5444), those with prediabetes (*n*=8246) and those without diabetes (*n*=5234) during a median follow-up of 2.8 years (RR 0.85). However, as participants with diabetes had two times more CV events than participants without diabetes during follow-up, in the group of alirocumab-treated participants, the subgroup with diabetes had a greater absolute risk reduction of the primary endpoint than the subgroup without diabetes (−2.3% vs −1.2%; *p*_interaction_=0.0019). Alirocumab did not increase the risk of NODM in participants without diabetes or those with prediabetes, and had no effect on HbA_1c_ or blood glucose levels in normoglycaemic individuals or those with prediabetes. In addition, a recent meta-analysis of 39 RCTs including 35,896 participants treated with alirocumab or evolocumab did not find any association between the use of these drugs and NODM (*p*=0.97) [23]. Finally, the open-label study of FOURIER, exploring the efficacy and safety of evolocumab over a median of 5 years (with a maximal exposure period of 8.4 years) confirmed that the number of cases of NODM in the evolocumab group was similar to that in the placebo group and did not increase over time [24].

Inclisiran, an siRNA targeting PCSK9 and administered twice yearly (after initial and 3 month doses), was approved by the European Medicines Agency (EMA) in 2020 and then by the FDA in 2021. In the Phase III ORION studies, the placebo-corrected percentage reduction in LDL-C with inclisiran was 50.6% [25]. A post hoc analysis of the Phase II study, ORION-1 [26], showed that inclisiran decreased LDL-C irrespective of diabetes status (in 67 participants with diabetes vs 415 participants without diabetes) [27]. In the ORION-11 study, including 203 primary prevention participants at high CV risk under statin therapy and among whom 132 had diabetes, there was a significant reduction in a range of circulating atherogenic lipoproteins, including LDL-C (43.7%), non-HDL-C (39.5%) and apolipoprotein (apo) B (35.8%) at day 510 [28] (Table 1). The safety reports in the Phase III studies revealed that inclisiran was well tolerated except for injection site reactions (mostly mild, none severe) and a modest excess of mild-to-moderate bronchitis [25]. Moreover, in the 4 year open-label ORION-3 study, which included 382 participants, among whom 87 had diabetes, there was a sustained reduction in LDL-C over the 4 years of exposure to inclisiran (averaged mean reduction in LDL-C was 44.2%) [29]. In this long-term study, 14% of participants had injection site reactions and 1% had a serious adverse event that was possibly related to the drug. Three CVOTs of inclisiran are ongoing (ORION-4 [NCT03705234] and VICTORION-1 and -2 Prevent [NCT05739383 and NCT05030428, respectively]).

In summary, these recently approved drugs targeting PCSK9 (antibodies and siRNA) are highly effective at reducing LDL-C and CV risk and are well tolerated, particularly in those with diabetes. At present, there are no clinical studies showing that these drugs cause new-onset diabetes or worsen diabetes, but longer exposure studies are needed.

#### Therapy targeting ATP citrate lyase: bempedoic acid

Bempedoic acid is an oral, once daily, first-in-class drug that inhibits ATP citrate lyase, an enzyme involved in cholesterol synthesis and located upstream of 3-hydroxy-3-methylglutaryl-coenzyme A (HMGCoA) reductase, the target of statins. Bempedoic acid was approved for use in adults by the FDA and EMA in 2020. In a recent analysis of four pooled Phase III trials including 3621 participants, among whom 678 had diabetes, exposure to bempedoic acid for a median of 1 year decreased LDL-C levels by 17.4–28.5% [30]. In a dedicated post hoc analysis of the same trials, bempedoic acid modestly but significantly reduced HbA_1c_ by 0.12% in those with diabetes and by 0.06% in those with prediabetes compared with placebo (Table 1). The annual rate of NODM among normoglycaemic individuals or those with prediabetes was not increased in the bempedoic acid group compared with the placebo group (0.3% vs 0.8% and 4.7% vs 5.9%, respectively) [31]. The efficacy of the drug for lowering LDL-C was the same according to glycaemic status. Moreover, a Mendelian randomisation study of genetic scores composed of inherited variants in the gene coding for ATP citrate lyase did not find any association between these scores and diabetes, contrary to *HMGCR*, *NPC1L1*, *PCSK9* and *LDLR* scores (genes encoding the targets of statins, ezetimibe and PCSK9 inhibitors) [32], but other studies are needed to confirm these results. The recent CLEAR CVOT, which included 13,970 statin-intolerant participants, demonstrated a significant decrease of 13% in the primary endpoint (composite of CV death, MI, stroke or coronary revascularisation) in the bempedoic acid group compared with the placebo group, with a median duration of follow-up of 40.6 months [33]. A large proportion of participants with diabetes were included in this study (*n*=6373, 45.6%), and there was no worsening of diabetes or NODM in those without diabetes treated with bempedoic acid compared with placebo. Longer follow-up studies are needed to confirm these results. The safety profile of bempedoic acid is acceptable, with an increased risk of gout or cholelithiasis (seen only in the CLEAR CVOT) and a small increase in serum creatinine, uric acid and liver enzymes.

In summary, bempedoic acid is effective at reducing LDL-C and CV risk and is quite well tolerated, regardless of glycaemic status, and has a neutral to positive effect on glycaemic variables in those with hypercholesterolaemia.

### Potential future therapies

#### Therapies targeting proprotein convertase subtilisin/kexin 9

Other emerging therapies targeting PCSK9 are in development: an adnectin (recombinant fusion protein of a PCSK9-binding domain and human serum albumin), namely lerodalcibep, which has been shown to decrease LDL-C levels by 60–70% with monthly s.c. injections [34], a vaccine against PCSK9 [35] and a gene editing approach using clustered regularly interspaced short palindromic repeats (CRISPR)–Cas9 technology [36]; both the vaccine and the gene editing approach are undergoing preclinical and clinical trials.

## Therapies mainly targeting triglycerides/HDL-cholesterol

### Historical treatments

Fibrates have been used for decades to reduce the risk of acute pancreatitis in severe hypertriglyceridaemia. Their use in the prevention of CV risk remains controversial. In the large FIELD trial in individuals with type 2 diabetes, fenofibrate failed to reduce the primary outcome of coronary events compared with placebo [37, 38]. In the ACCORD CVOT, the combination of fenofibrate and simvastatin did not reduce the rate of fatal CV events, non-fatal MI or non-fatal stroke compared with placebo in participants with type 2 diabetes [39]. Prespecified subgroup analyses suggested a possible benefit for men and for participants with both hypertriglyceridemia ≥2.30 mmol/l and low HDL-C ≤0.88 mmol/l at baseline. In the recent PROMINENT CVOT including individuals with type 2 diabetes with mild-to-moderate hypertriglyceridemia, low levels of HDL-C and well-controlled levels of LDL-C, pemafibrate, a selective peroxisome proliferator-activated receptor α modulator, did not reduce the risk of CV events. At 4 months, pemafibrate resulted in mean favourable changes in TG (−26.2%) and HDL-C (+5.1%) levels, a neutral change in non-HDL-C levels (−0.2%) and unfavourable changes in LDL-C (+12.3%) and apoB (+4.8%) levels compared with placebo [40]. A meta-analysis of 18 prospective RCTs assessing the effects of fibrates on CV outcomes compared with placebo showed that fibrate therapy produced a significant 10% RR reduction in major cardiovascular events and a 13% RR reduction in coronary events, but had no benefit on stroke, all-cause mortality, cardiovascular mortality, sudden death or non-vascular mortality. There was no difference in the results for coronary events between those with diabetes and those without diabetes [41]. Another meta-analysis of five RCTs of fibrate (ACCORD, FIELD, BIP, HHS and VA-HIT) showed that fibrate treatment produced a significant 35% RR reduction in coronary heart disease in the subgroup of participants with atherogenic dyslipidaemia (high TG levels and low HDL-C levels), which is common in diabetes [42]. These data have led to divergent guidelines. In ADA standards of care in diabetes, statin plus fibrate combination therapy is generally not recommended [43], whereas in European Society of Cardiology (ESC)/European Atherosclerosis Society (EAS) guidelines fenofibrate or bezafibrate may be considered in combination with statins for primary prevention or in high-risk individuals at LDL-C target levels with TG levels >2.3 mmol/l [44]. We consider that fenofibrate may be considered in combination with statins in high- or very-high-risk individuals at LDL-C target levels with TG levels >2.3 mmol/l and HDL-C levels <1 mmol/l in men or <1.3 mmol/l in women.

Considering the apparent contrasting effects of fibrates, new-generation therapies are needed to improve the lipid abnormalities seen in atherogenic dyslipidaemia.

### Newly approved treatments

#### Omega-3 fatty acids

Omega-3 fatty acids can be used at pharmacological doses (2–4 g/day) to lower TG levels. A meta-analysis of RCTs including 77,917 people treated with docosahexaenoic acid (DHA) plus eicosapentaenoic acid (EPA) at doses between 226 and 1800 mg/day showed a neutral effect on mortality and CV events, including in the subgroup with diabetes [45]. Two more RCTs have confirmed the inability of omega-3 supplementation (1 g/day and 4 g/day of EPA and DHA, respectively) to reduce CV events in participants with diabetes without evidence of CV disease [46] and in statin-treated participants at high CV risk (70% of people with diabetes) [47]. However, the REDUCE-IT study, which included 8179 secondary prevention participants or participants with diabetes at high CV risk who had been receiving statins and had TG levels between 1.5 and 5.6 mmol/l and LDL-C levels between 1 and 2.6 mmol/l, showed that high-dose treatment with 4 g/day of icosapent ethyl (IPE) was associated with significant reductions of 25% in the primary endpoint (CV death, non-fatal MI, non-fatal stroke, coronary revascularisation or unstable angina), 20% in CV mortality and 28% in fatal and non-fatal fatal stroke and a non-significant reduction of 13% in total mortality [48]. Median changes between the last visit and baseline in the IPE group were −14.1% for TG, −3% for HDL-C, −8.6% for non-HDL-C, −7.4% for LDL-C and −6.7% for apoB (Table 1). However, lowering of TGs is unlikely to be the full explanation for the CV benefit of IPE, as benefits were similar irrespective of the degree of TG lowering in those receiving IPE. The median follow-up in this study was 4.9 years.

Currently, IPE 2×2 g/day is recommended in high-risk (or above) individuals with TG levels between 1.5 and 5.6 mmol/l (135 and 499 mg/dl) despite statin treatment, to reduce CV risk [42, 43].

#### Therapy targeting apoC-III

ApoC-III is currently recognised as a key regulator of TRL metabolism and mediates its effects through both lipoprotein lipase (LPL)-dependent and LPL-independent mechanisms (Fig. 1). The plasma apoC-III concentration is strongly positively correlated with the plasma TG concentration and is increased in insulin-resistant states. Importantly, hyperglycaemia in individuals with type 1 or type 2 diabetes is associated with increased apoC-III levels [49]. Elevated apoC-III has been postulated to contribute to atherogenic dyslipidaemia through the impairment of TRL and HDL metabolism [50, 51]. Volanesorsen, an antisense oligonucleotide targeting hepatic apoC-III, was first studied in the APPROACH and COMPASS Phase III trials in individuals with familial chylomicronaemia syndrome (FCS) [52] and in individuals with multifactorial chylomicronaemia syndrome (MCS), including 40% of participants with diabetes [53]. In the latter study, volanesorsen reduced mean TG levels by 71.2% and mean non-HDL-C levels by 27.3% and increased mean HDL-C levels by 61.2% and mean LDL-C levels by 95.5% from baseline to 3 months, with no change in apoB levels (Table 1). In the BROADEN Phase II/III trial, which included individuals with familial partial lipodystrophy and concomitant hypertriglyceridaemia and diabetes, volanesorsen reduced mean TG levels by 88% from baseline to 3 months and the hepatic fat fraction by 52% from baseline to 12 months, with no change in HbA_1c_ [54]. The main side effects of volanesorsen were injection site reactions and thrombocytopenia.

In summary, volanesorsen is very effective at reducing TG levels and should be very helpful in reducing the risk of acute pancreatitis; however, because of its side effects, particularly thrombocytopenia, volanesorsen has not been approved by the FDA and further CVOTs are needed.

#### Therapy targeting angiopoietin-like 3

Angiopoietin-like 3 (ANGPTL3), a protein that is exclusively synthesised in the liver, may increase VLDL secretion and inhibits the activity of two extracellular lipases: LPL, leading to a decrease in TRL catabolism, and endothelial lipase, leading to decreased LDL hepatic uptake and the abrogation of HDL phospholipid catabolism, raising HDL-C levels [50] (Fig. 1). In 2021, the first-in-class human anti-ANGPTL3 monoclonal antibody, evinacumab, was approved by the EMA and FDA for use in homozygous familial hypercholesterolaemia. Recently, because of the high efficacy of evinacumab to decrease TG levels, several studies have been carried out in individuals with hypertriglyceridaemia. Two Phase II studies in participants with TG >1.7 mmol/l but ≤5.1 mmol/l and LDL-C ≥2.6 mmol/l (but without diabetes) were randomised to s.c. or i.v. evinacumab at different doses compared with placebo. The maximal difference between evinacumab and placebo was −88% for TG, −28% for HDL-C, −35% for non-HDL-C and −25% for LDL-C (Table 1). Evinacumab was well tolerated with no serious adverse events [55].

In summary, evinacumab is very effective at reducing TG levels in people with MCS and should be very helpful at reducing the risk of acute pancreatitis and reducing LDL-C levels. It is well tolerated but further CVOTs are needed.

### Potential future therapies

#### Therapy targeting apoC-III

Because of the main side effects of volanesorsen (injection site reactions and thrombocytopenia), olezarsen, an *N*-acetyl-galactosamine (GalNac)-conjugated antisense oligonucleotide that binds more specifically to apoC-III in the liver, was designed. Olezarsen has enhanced safety and tolerability compared with volanesorsen as it is used at a lower dose and injection volume with less frequent dosing. A randomised, double-blind, placebo-controlled, dose-ranging study of olezarsen was conducted in 114 individuals at high risk for or with established CV disease (67.5% with type 2 diabetes) and with fasting serum TG levels of 2.26–5.65 mmol/l [56]. Treatment with olezarsen resulted in mean per cent TG reductions from baseline of 23–60%. HDL-C increased significantly (from 12% to 42%) in each olezarsen dose group (10 or 50 mg every 4 weeks, 15 mg every 2 weeks or 10 mg every week), non-HDL-C decreased significantly (from 15% to 24%) and apoB decreased significantly (from 10% to 16%) but not in all olezarsen groups, and LDL-C increased significantly (23%) in only one olezarsen group. Regarding side effects, there were no platelet count, liver or renal function changes in any of the olezarsen groups and the most common adverse event was mild erythema at the injection site. Several Phase III studies of olezarsen are in progress in FCS (NCT04568434) MCS (NCT05079919 and NCT05552326) and individuals with hypertriglyceridaemia and at high risk for or with established CV disease (NCT05610280). In the context of the dual effect of olezarsen—improvement in some lipid levels (reduction in TG and non-HDL-C and increase in HDL-C) but deterioration in others (increase in LDL-C and no significant change in apoB for all treated groups)—CVOTs will be very important to assess the effect of the drug on ASCVD risk. Indeed, whereas, in general, LDL-C, non-HDL-C and apoB are very highly correlated and provide very similar information about ASCVD risk, under certain circumstances, including in people with diabetes, LDL-C measurement is less reliable. In this case, apoB, which provides an accurate estimate of the total concentration of atherogenic particles, can be the preferred measurement to further refine the estimate of ASCVD risk that is modifiable by lipid-lowering therapy [43].

Moreover, injection of the hepatocyte-targeted GalNac-conjugated siRNA ARO-APOC3 is currently under development in Phase III studies (NCT04998201, NCT04720534 and NCT05089084). A monoclonal antibody approach to lowering of apoC-III has also been described [57].

#### Therapy targeting ANGPTL3

The development programme for vupanorsen, a (GalNac)-conjugated antisense oligonucleotide targeted to the liver that selectively inhibits ANGPTL3 protein synthesis, was discontinued in 2022. Indeed, the magnitude of decreases in TG and non-HDL-C levels observed in the TRANSLATE-TIMI 70 study (Phase IIb) did not support continuation of the clinical development programme for CV risk or severe hypertriglyceridaemia. Vupanorsen was also associated with dose-dependent increases in liver fat, and higher doses were associated with elevations in the liver enzymes alanine aminotransferase and aspartate aminotransferase [58].

The effects of a single or repeat s.c. doses of the (GalNac)-conjugated siRNA ARO-ANG3, which degrades *ANGPTL3* mRNA in healthy volunteers and in individuals with hepatic steatosis, were recently described. In the cohort of participants with hepatic steatosis, which is common in type 2 diabetes, repeat dosing of 200 mg resulted in mean reductions in TG (−42%), non-HDL-C (−37%), LDL-C (−36%), apoB (−20%) and HDL-C (−57%) levels at day 113 compared with baseline. The treatment was well tolerated [59]. More work is needed to confirm these results.

#### Fibroblast growth factor 21 agonists

Fibroblast growth factor 21 (FGF21) is an endogenous stress hormone, a member of the FGF family, and is primarily produced by the liver. It binds to and activates FGF receptors and regulates lipid and glucose metabolism and energy expenditure. Several FGF21 analogues are in development. Most of the Phase I and II trials have been dedicated to type 2 diabetes or included a large proportion of participants with type 2 diabetes. The results of these studies vary according to drug, dose and duration of treatment. However, generally, reductions of up to 69% in TG, 30% in LDL-C, 34% in non-HDL-C and 25% in apoB and an increase of up to 61% in HDL-C are observed [60, 61]. In the study of non-alcohol-related steatohepatitis (NASH) [60], the liver fat fraction was reduced after treatment with the FGF21 analogue. Glucose control was not systematically improved, but longer studies are needed. The different drugs are well tolerated and the most common adverse events are related to gastrointestinal disturbances. Phase III studies are ongoing with pegozafermin (NCT05852431) and efruxifermin (NCT06161571).

#### Therapy targeting cholesteryl ester transfer protein

Cholesteryl ester transfer protein (CETP) inhibitors lead to the greatest elevations in HDL-C levels (Fig. 1), but the results of clinical trials have been disappointing [62]. A recent randomised Phase II trial in dyslipidaemic participants using a new CETP inhibitor (obicetrapib) in combination with background high-intensity statin treatment found that, after 8 weeks, there was a significant decrease in median TG (−11% only with obicetrapib 5 mg), non-HDL-C (by up to −44%), LDL-C (by up to −51%) and apoB (by up to −30%) levels and an increase in median HDL-C levels (by up to 165%) [63]. Two meta-analyses found a significant reduction in the risk of new-onset diabetes of 12% [64] and 16% [65] in the CETP inhibitors group compared with the placebo group; glycaemic measures were also significantly improved in those with and without diabetes across most trials [65]. The results of the CVOT PREVAIL (NCT05202509) are eagerly awaited.

## Therapies mainly targeting lipoprotein (a)

### Potential future therapies

Lipoprotein (a) (Lp(a)) is an inherited established CV risk factor, conferring an additional CV risk in people with diabetes. It is composed of an apo(a), which has pro-inflammatory and pro-thrombotic properties, linked to an LDL-like particle carrying cholesterol and oxidised phospholipids. Extended genetic and observational studies have demonstrated that a high Lp(a) concentration is causal for ASCVD, aortic valvular stenosis and cardiovascular and all-cause mortality [66]. As high Lp(a) concentrations are genetically determined and poorly influenced by the environment, only one measurement in life in adults is recommended. The EAS expert panel recommends more intensive CV risk management in those with high Lp(a) concentrations and a high baseline risk [66]. It is recommended that Lp(a) levels are measured at least once in individuals with diabetes to personalise the CV risk estimation [66, 67]. To date, there is no effective and recommended therapy targeting Lp(a). Three emerging gene silencing therapies targeting apo(a) hepatic production are in development (Fig. 1); the most advanced is pelacarsen (antisense oligonucleotide), followed by olpasiran (siRNA) and SLN360 (siRNA) [68]. However, there is a question about the potential of new Lp(a)-lowering drugs to increase the risk of diabetes. Indeed, recent observational and epidemiological studies have shown an association between very low Lp(a) levels and an increased risk of type 2 diabetes [69–71]. A meta-analysis of available studies found a 38% increased risk of diabetes in the bottom quintile of Lp(a) levels (threshold <3–5 mg/dl) compared with the upper quintile (threshold >27–55 mg/dl) [66]. Mendelian randomisation studies yielded contradictory results on this association, and the mechanisms underlying the association are unknown [72]. The results of the ongoing CVOTs on pelacarsen and olpasiran will bring insight to the association between very low Lp(a) concentrations and the risk of diabetes, the impact of the drug on CV events and the risk–benefit ratio in treated individuals.

## Conclusions

CVD is the leading cause of death in diabetes; despite significant progress in the prevention and the management of CVD over the last few decades, the current challenge remains to reduce CV risk. Regarding the management of lipid abnormalities in diabetes, lowering LDL-C levels is usually the first goal, with the cornerstone of treatment being statins, which may be combined with ezetimibe and/or PCSK9 inhibitors. Beyond controlling LDL-C levels, it is necessary to improve the lipid abnormalities that are responsible for residual CV risk: elevated Lp(a) levels and atherogenic dyslipidaemia. The results of clinical trials of fibrates and omega-3 have been disappointing, with the exception of trials on high-dose IPE, mainly targeting TG level, or CETP inhibitors, mainly targeting HDL-C levels. New drugs under development with the aim of decreasing LDL-C, TG or Lp(a) levels are promising to reduce the burden of CV risk, particularly in those with diabetes, or the risk of acute pancreatitis, in the case of TG-lowering-treatments. These drugs have favourable, unfavourable or neutral effects on the risk of new-onset diabetes or glycaemic control. Further studies are needed to confirm the efficacy of these new drugs and monitor their potential side effects, particularly with regard to glycaemic control. A summary of next-generation therapies in the field of dyslipidaemia in individuals with diabetes is provided in the text box.

### Supplementary Information

Below is the link to the electronic supplementary material.

Supplementary file1 (PPTX 293 KB)

## Abbreviations

ANGPTL3: Angiopoietin-like 3
Apo: Apolipoprotein
ASCVD: Atherosclerotic CVD
CETP: Cholesteryl ester transfer protein
CVOT: Cardiovascular outcome trials
DHA: Docosahexaenoic acid
EMA: European Medicines Agency
EPA: Eicosapentaenoic acid
FCS: Familial chylomicronaemia syndrome
FDA: Food and Drug Administration
FGF21: Fibroblast growth factor 21
GalNac: *N*-acetyl-galactosamine
HDL-C: HDL-cholesterol
IPE: Icosapent ethyl
LDL-C: LDL-cholesterol
Lp(a): Lipoprotein (a)
LPL: Lipoprotein lipase
MCS: Multifactorial chylomicronaemia syndrome
MI: Myocardial infarction
NODM: New-onset diabetes mellitus
PCSK9: Proprotein convertase subtilisin/kexin 9
TG: Triglycerides
TRL: Triglyceride-rich lipoprotein
