# Supplementary material for: Hyperlipidaemia in diabetes: are there particular considerations for next-generation therapies?
Source: Diabetologia. 2024 Feb 20;67(6):974–84. doi: 10.1007/s00125-024-06100-z (PMC11058750; doi:10.1007/s00125-024-06100-z)
Supplement: Supplementary file 1 — Supplementary file1 (PPTX 293 KB) [file 125_2024_6100_MOESM1_ESM.pptx]

## Slide 1
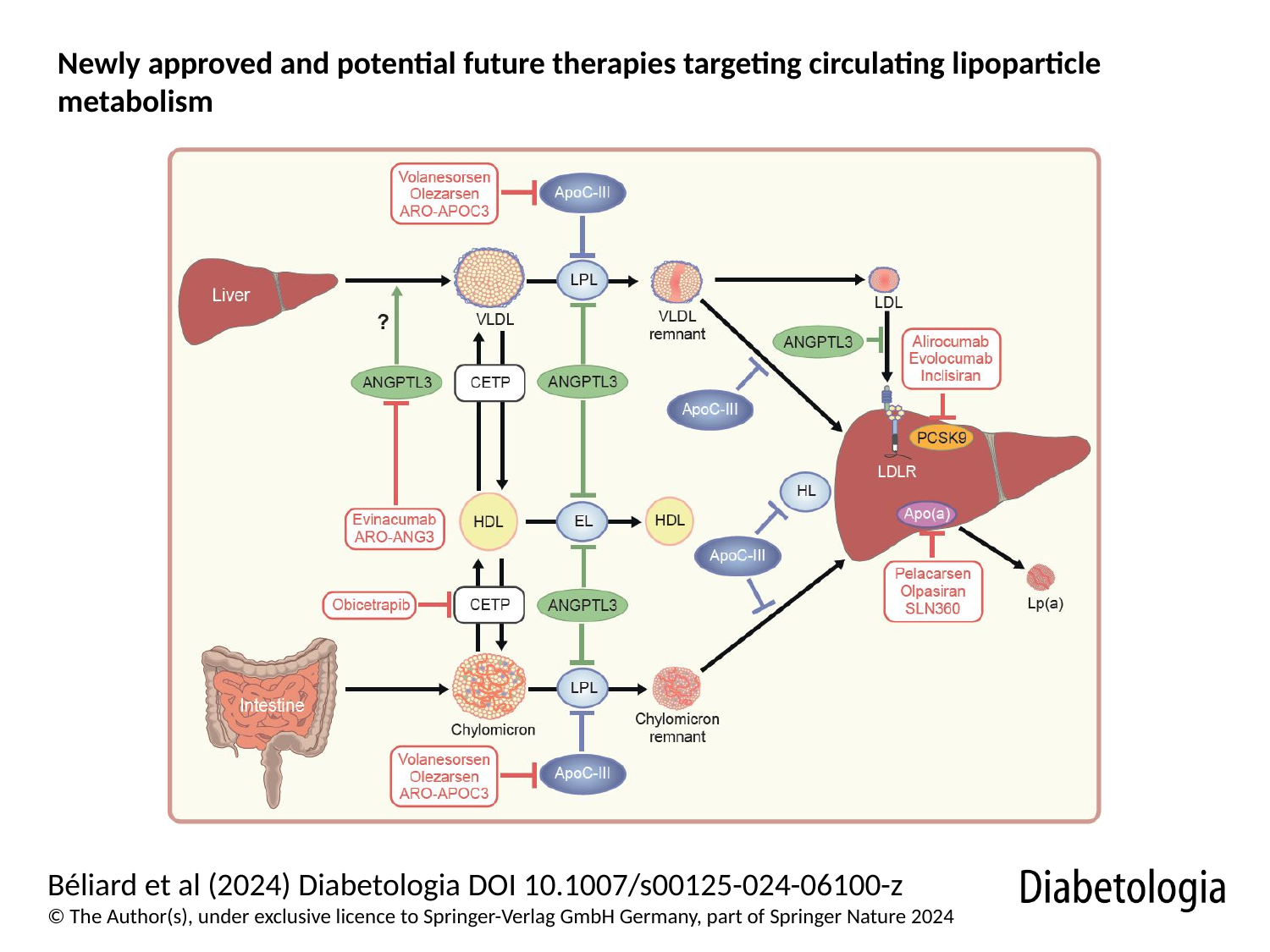

Newly approved and potential future therapies targeting circulating lipoparticle metabolism
Béliard et al (2024) Diabetologia DOI 10.1007/s00125-024-06100-z
© The Author(s), under exclusive licence to Springer-Verlag GmbH Germany, part of Springer Nature 2024
